# Associations between depressive symptoms and 5-year subsequent work nonparticipation due to long-term sickness absence, unemployment and early retirement in a cohort of 2,413 employees in Germany

**DOI:** 10.1186/s12889-023-17090-9

**Published:** 2023-11-03

**Authors:** Uwe Rose, Norbert Kersten, Dagmar Pattloch, Paul Maurice Conway, Hermann Burr

**Affiliations:** 1 Federal Institute for Occupational Safety and Health (BAuA), Nöldnerstr. 40/42, D-10317 Berlin, Germany; 2 Department of Psychology, University of Copenhagen, Øster Farimagsgade 2A, København, 1353 Denmark

**Keywords:** Longitudinal study, Depression, Sickness absence, Employment, Disability pension, Work participation

## Abstract

**Background:**

We examined the association of depressive symptoms with subsequent events – and duration thereof – of work nonparticipation (long-term sickness absence, unemployment and early retirement).

**Methods:**

We employed a 5-year cohort from the Study on Mental Health at Work (S-MGA), based on a random sample of employees subject to social contributions aged 31–60 years in 2012 (N = 2413). Depressive symptoms were assessed at baseline through questionnaires, while work nonparticipation was recorded in follow-up interviews. Associations of depressive symptoms with subsequent events of work nonparticipation were examined in two-part models, with events analysed by logistic regressions and their duration by generalized linear models.

**Results:**

Medium to severe depressive symptoms were associated with events of work nonparticipation (males Odds Ratio [OR] = 3.22; 95% CI = 1.90–5.45; females OR = 1.92; 95% CI = 1.29–2.87), especially with events of long-term sickness absence in both genders and events of unemployment in males. Mild depressive symptoms were also associated with events of work nonparticipation (males OR = 1.59; 95% CI = 1.19–2.11; females OR = 1.42; 95% CI = 1.10–1.84). Among those experiencing one or more events, the duration of total work nonparticipation was twice as high among males [Exp(β) = 2.06; 95% CI = 1.53–2.78] and about one third higher [Exp(β) = 1.38; 95% CI = 1.05–1.83] among females with medium to severe depressive symptoms.

**Conclusions:**

The present study focuses on both events and duration of work nonparticipation, which are both critical for examining societal consequences of depressive symptoms. It is key to regard also mild depressive symptoms as a possible risk factor and to include different types of work nonparticipation.

## Background

This paper deals with associations between depressive symptoms and work nonparticipation. The opposite of work nonparticipation has been conceptualized as “[…] the capability and/or opportunity to participate in the workforce, fulfilling one’s work role” [1].

Over the last two decades, the proportion of registered sickness absence events attributed to mental health problems has increased in Germany [2]. Absence spells lasting less than six weeks account for approximately half of all days of absence due to sickness [3]. The proportion of disability pensions due to mental disorders has also increased from 19 to 43% over 22 years [4]. These figures point to the substantial role that mental health plays in terms of individual (reduced income) as well as direct (health problems) and indirect (taxes, support payments) costs for the social security system [5]. Mental health impairments can be linked to both short- and long-term forms of work nonparticipation. While sickness absence is typically a temporary state, early retirement (ER) and disability pension are – with a few exceptions – permanent [6]. In addition to sickness absence and ER before statutory retirement, unemployment (UE) should be regarded as a further type of work nonparticipation [6, 7]. Both temporary and permanent states of work nonparticipation were previously found in association with productivity losses [5].

In most countries, cases of registered sickness absence and disability pension are attributed to one or more diagnoses. This attribution is the result of a decision-making process that takes not only clinical but also administrative and regulatory aspects into account [8, 9]. Thus, the diagnoses reported in administrative registries as the underlying reason for sickness absence or disability pension might not necessarily align with the real associations of these diagnoses with sickness absence and disability pension. This is reflected, for example, by the increasing number of events of sickness absence and disability pension due to mental health problems that occurred in recent decades, which stands in contrast with the rather stable prevalence of mental disorders in Europe [10, 11]. Therefore, it might be that administrative statistics shed light on the predictive role of mental health in relation to sickness absence and disability pension only to a limited degree. To bridge this gap, epidemiological evidence on the possible causal relationship between mental health conditions and work (non)participation is needed that relies on longitudinal studies examining depressive disorders or depressive symptoms as predictors of sickness absence, disability pension, ER, and UE.

Among mental health conditions, depression is one of the most disabling [11]. Usually, a diagnostic interview is the basis for classifying an individual in a discrete depression category [12, 13], whereas depression as a continuum is traditionally assessed by using symptom inventories administered via paper and pencil questionnaires [14, 15].

To date, the evidence on depressive symptoms as a predictor of work (non)participation based on representative samples and cohort study designs is limited. Most existing studies examined events of work nonparticipation but did not take their duration into account. A few studies suggested an association between depressive symptoms and sickness absence. Specifically, the Household Income and Labour Dynamics (HILDA) Survey in Australia [16] and the Maastricht Cohort Study in The Netherlands [17] showed that increased levels of depressive symptoms were associated with higher incidence rate ratios or hazard rates of short- and long-term sickness absence. The rates were slightly higher among males in the first study, while there were no gender differences in the latter study. A similar result was observed in the Danish National Working Environment Survey (DANES), which linked self-reported depressive symptoms at baseline to 60-week follow-up data on long-term sickness (≥ 4 weeks) derived from a nationwide register, with no stratification by gender [18]. However, long-term sickness absence is only one type of exit from paid work. In previous studies, depressive symptoms were also associated with forms of work nonparticipation other than sickness absence [19]. For example, in a study based on the Survey of Health, Ageing and Retirement in Europe (SHARE), depressive symptoms were associated with disability benefits and UE [20]. For males and females with depressive symptoms, the hazard rates for disability benefits were HR = 3.29 (95% CI = 1.99–5.46) and HR = 2.10 (95% CI = 1.17–3.77), respectively, while the association between depressive symptoms and UE was not significant (males: HR = 1.55 (95%-CI = 0.94–2.57); females: HR = 1.14; 95%-CI = 0.73–1.76). In their systematic review on the link between poor health and exit from paid work, Rijn et al. [19] found associations between indicators of poor (mental) health and later disability pension, UE and ER. In sum, the current state of the art suggests that, with only a few exceptions [20, 21], there is to date limited knowledge about the association between depressive symptoms and different types of work nonparticipation. This limits the current understanding of whether the association between depressive symptoms and long-term sickness absence (LTSA; i.e., the most frequently investigated outcome) [16–18, 21, 22] is of a similar or a different size than their association with UE [20, 21] or ER [17, 23]. For instance, in health economic analyses this knowledge gap prevents one from summarising different types of work nonparticipation into single measures.

Events of work nonparticipation can be recorded both in terms of occurrence and their duration. However, to date longitudinal studies have predominantly captured event occurrence by treating events as dichotomous outcomes. While event-related analysis is important for examining the general causal relationship between depressive symptoms and different outcomes, such as permanent exit from paid employment, it does not capture its duration, which allows for the assessment of the associated productivity loss. Only a few studies have previously attempted to model these time aspects [17, 21, 24–26].

In addition, while most available studies have stratified the analyses by gender [16, 17, 20–22], only one [17] found significant indications of gender differences in the association between mental health and work nonparticipation. However, none of these studies employed statistical tests to examine interaction by gender.

Based on the above-mentioned considerations, in the present study we aim to investigate the associations of depressive symptoms with two aspects of work nonparticipation, that is, occurrence and duration of events of work nonparticipation. Based on the aforementioned literature review [20, 21], we do assume that the associations between depressive symptoms and work participation will differ between males and females.

## Methods

### Sample

**Fig. 1:**
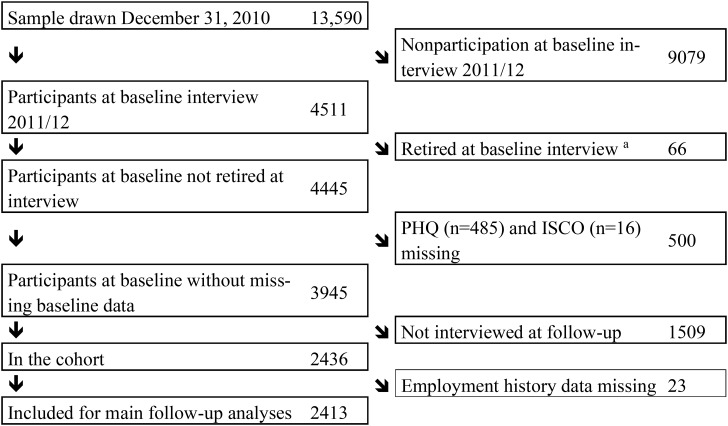
Flow diagram of participation in the S-MGA at baseline (2011/12) and from baseline to follow-up (2017). ^a^ Between the sampling and interview, there was a time lag of an average of 13 months (range 11-17)

We analysed a cohort based on the Study on Mental Health at Work (in German: S-MGA), using both the baseline (2011/12) and follow-up (2017) measurements. This study was initiated by the Federal Institute of Occupational Safety and Health in collaboration with the Institute of Employment Research (IAB). The latter incorporates data on all employees in Germany subject to social security contributions in the Integrated Employment Biographies (IEB) register, which covers more than 80% of the working population in Germany. Employees subject to social insurance contributions as of 31 December 2010 were registered in the IEB; civil servants, self-employed individuals and freelancers were excluded by this definition. The S-MGA includes cohorts of individuals born between 1951 and 1980. To facilitate face-to-face interviews, a two-stage cluster sampling procedure was applied to a random sample of 206 municipalities in Germany proportionately stratified by region and population, followed by a random gross sample of 13,590 addresses drawn within selected municipalities [27]. A flow-chart of participation is shown in Fig. 1. Altogether, 4511 individuals participated in a computer-assisted personal interview (CAPI) at baseline. The follow-up took place in 2017 (n = 2640). Since there was a gap of an average of 13 months between the sampling and the interview dates, in which participants could retire, 66 participants were excluded from the analysis. In addition, 500 participants from the baseline measurement were excluded because they presented missing values on depressive symptoms or skill level (see the subsections ‘Independent variables’ and ‘Covariates’ below). As for the cohort, the largest loss was due to 1509 respondents not taking part in the follow-up survey after having participated at baseline, and to 23 respondents for whom the employment biography could not be established at follow-up. The final sample included in the present analyses consisted of a cohort of 2413 individuals (n = 1168 males and n = 1245 females) that participate in both measurements. This corresponds to a participation in the cohort study of 18% (Fig. 1). Based on comparisons with the sampling frame of the study, participation in the cohort was independent of gender, while it was somewhat associated with age (20% among those aged 55–60, 15% among those aged 31–36) and skill level (24% among professionals and managers and 14% among unskilled workers) [28].

All information was obtained in the respondents’ home, mainly through the CAPI, whereas sensitive information about depressive symptoms was gathered through paper-and-pencil questionnaires that were completed without the interviewer being present.

### Independent variables

We categorized depressive symptoms into three categories by means of the Patient Health Questionnaire (PHQ-9) [29]: ‘medium to severe’ (≥ 10) [30], ‘mild’ (5–9), and ‘none’ (0–4). The PHQ-9 yields a sum score based on nine items which were preceded by the following question: ‘Over the last 2 weeks, how often have you been bothered by any of the following problems?` The nine items were: ‘Little interest or pleasure in doing things’, ‘Feeling down, depressed or hopeless’ ‘Difficulty falling asleep or sleeping or increased sleep’, ‘Tiredness or feeling unable to have energy’, ‘Decreased appetite or excessive need to eat’, ‘Bad opinion of yourself’, ‘Difficulty concentrating on something’, ‘Slowed speech/movement or restlessness (“fidgety”)’, ‘Thoughts that you would rather be dead or want to self-inflict pain’. The response options were: ‘Not at all’ (0), ‘Several days’ (1), ‘More than half the days’ (2) and ‘Nearly every day’ (3). The score for depressive symptoms is computed as the sum of all 9 items. Cronbach’s Alpha was .82 and the inter-item correlations were between .22 and .51. Based on previous analyses performed by authors of this study, the distribution of the PHQ values was positively skewed, which is due to our baseline sample being healthier than the general population in the same age range.

### Outcomes

We considered different forms of work nonparticipation, that is, LTSA, UE and ER, occurring between baseline and follow-up. A few events of work nonparticipation for which participants did not report the specific type were also considered but not examined separately due to their low incidence (Table 1). Each event was measured in terms of the year and month it started and ended. Regarding LTSA, respondents were asked to only report events lasting at least 6 weeks. This restriction did not apply to UE and ER. As in our analyses we treated the various forms of work nonparticipation as mutually exclusive events, we considered only one event as reported by participants in a given year and month. However, events could take place consecutively over time; for instance, first an event of sickness absence followed by an event of ER. Since respondents were allowed to report LTSA events which overlapped with events of UE and ER, for the present analyses we prioritized UE and ER, respectively, over LTSA. Therefore, any events of LTSA that overlapped with UE and ER were not considered.

**Table 1:** Sample characteristics for the dependent variables

	Males (n = 1168)		Females (n = 1245)	
	N	%	N	%
Follow-up months total	73,079	100	77,913	100
Total work nonparticipation (TWNP)	5080	7	5738	7
Long-term sickness absence (LTSA)	1666	2	2186	3
Unemployment (UE)	1574	2	1530	2
Early retirement (ER)	1709	2	1707	2
Other work nonparticipation	131	0	315	0
Median TWNP, months ^a^ (by subject)	6		6	
Mean TWNP, months ^a^ (by subject)	13		13	

^a^ Among those experiencing at least one event of work nonparticipation

We also calculated an index, labelled total work nonparticipation (TWNP), that included all events of LTSA, ER, and UE, and other types of work nonparticipation that could not be ascribed to any of these forms.

### Covariates

We considered gender, age at baseline, skill level and employment status as covariates. In line with previous studies [16, 17, 20–22], we used gender as stratification variable. Age at baseline was categorized into three decades (31–40, 41–50, 51–60). The skill level of the job was based on the International Standard Classification of Occupations (ISCO 08) of the International Labour Office and categorized into four groups: unskilled workers (ISCO groups 9), skilled workers (4–8), semi-professionals (3), and professionals (1,2). In the analyses, the most frequent group (‘skilled workers’) was used as reference. The variable “in paid employment at baseline interview” was treated as dichotomous (‘no’ vs. ‘yes’). We did not include indicators such as income and education as covariates. We did so because in our data skill level correlated with both education (Kendall Tau _b_ = 0.48) and income (categorized in quintiles; Kendall Tau _b_ =0.35) and introducing them in the analyses would have led to less stable regression coefficients. We prioritized skill level as education was a less proximal indicator of socio-economic status in relation to the time the present study was carried out. Moreover, income is more biased than skill level in relation to public/private employment (a number of skilled and academic workers in the public sector have lower income in the public than in the private sector).

### Statistical analysis

The number of months as outcome is a semicontinuous variable that takes exclusively non-negative integer values and is zero-inflated (i.e., a large stack of data points concentrates around zero as the lower bound). Therefore, the distribution appears as a mixture of zeros and positively skewed non-zero values. A common regression analysis or multiple regression analysis cannot be applied to such a distribution because assumptions such as normality and homoscedasticity are not met. Thus, we decided to use two-part models to analyse the present data.

The likelihood function $l$ of a two-part model [31, 32], with a variance parameter *σ*, takes the following form:

$$l\left({\varvec{\beta }}_{1},{\varvec{\beta }}_{2},\sigma \right)=\left[\prod _{\left\{i:{y}_{i}=0\right\}}P({y}_{i}=0|{\varvec{x}}_{1,i}^{ },{\varvec{\beta }}_{1})\right]\left[\prod _{\left\{i:{y}_{i>0}\right\}}P({y}_{i}>0|{\varvec{x}}_{1,i}^{ },{\varvec{\beta }}_{1}\left)f\right({y}_{i}|{\varvec{x}}_{2,i}^{ },{\varvec{\beta }}_{2},\sigma)\right]$$

${\varvec{\beta }}_{1}$and ${\varvec{\beta }}_{2}$ are two sets of regression parameters to be estimated in the model. $f$ is a density function specified in the concrete case. This likelihood function has only one global maximum, which can be decomposed into two parts, each of which can be maximized separately. First, the logistic regression part with parameters ${\varvec{\beta }}_{1}$ was used to model zero and non-zero values. Second, conditional on all non-zero values, generalized linear models (GLMs) with a gamma distribution and a log-link function were performed to model the non-zero values with parameter ${\varvec{\beta }}_{2}$.

In other words, the logistic regression component of the two-part model was used to model the event-related data. Odds ratios (OR) were calculated to estimate the risk of at least one event of nonparticipation between baseline and follow-up. Conditional on all non-zero events, GLM is used to model the number of months of work nonparticipation expected for these events. The estimates of a GLM with a logarithmic link function are expressed in terms of Exp(β), which is similar to an Odds Ratio (OR) derived from a logistic regression. Exp(β) and OR operate as multiplier. The predictive value, i.e., the number of months, is calculated by multiplying the exponential function of the intercept (Exp (β_0_)) by the exponential functions of the regression coefficients. In the GLM analyses, we only considered participants who reported at least one event of work nonparticipation.

As we expected that depressive symptoms were conditional of gender, in an additional analysis pooling together males and females, we tested if gender interacted with depressive symptoms by entering an interaction term (depressive symptoms*gender) in both the logistic and GLM regressions.

In all analyses, we calculated both crude estimates and estimates adjusted for the covariates. All statistical analyses were performed using IBM® SPSS® Statistics, version 27.01.0.

## Results

### Sample description

Tables 2 and 1 show the sample characteristics in both genders. The total follow-up time was 73,079 months for males and 77,913 months for females. The mean follow-up time was 63 months in both genders.

**Table 2:** Sample characteristics for the independent variable and the covariates

		Males (n = 1168)		Females (n = 1245)	
		N	%	N	%
Age at baseline					
	31–40	278	24	300	24
	41–50	487	42	518	42
	51–60	403	34	427	34
Skill level at baseline					
	Unskilled (9)	47	4	93	8
	Skilled (4–8)	542	46	456	37
	Semi-professional (3)	253	22	421	34
	Professional (1,2)	326	28	275	22
Paid employment at baseline					
	No^a^	38	3	73	6
	Yes	1130	97	1172	94
Depressive symptoms at baseline					
	None (< 5)	777	67	693	56
	Mild (5–9)	320	27	432	35
	Medium to severe (≥ 10)	71	6	120	10

^a^ E.g., parenthood, further education, UE at baseline

### Depressive symptoms and events of work nonparticipation

Tables 3 and 4 show, for males and females respectively, the OR adjusted for age, skill level, UE at baseline and the corresponding 95% confidence intervals (95% CI).

**Table 3:** Association between depressive symptoms at baseline and work nonparticipation at follow-up among males (n = 1168)

Outcome	Model 2^a^				
	Depressive symptoms				
	No (N = 777)	Mild (5–9) (n = 320)		Medium to severe (≥ 10) (n = 71)	
	OR	OR ^a^	95% CI	OR^a^	95% CI
TWNP	1	1.59	1.19–2.11	3.22	1.90–5.45
LTSA	1	1.48	1.08–2.02	2.96	1.76–4.97
UE	1	1.67	0.98–2.87	5.17	2.58–10.36
ER	1	1.72	1.01–2.92	1.20	0.47–3.06

TWNP: Total work nonparticipation; LTSA: Long-term sickness absence

UE: Unemployment; ER: Early retirement

^a^ Adjusted for age, ISCO-skill level, and paid employment at baseline

**Table 4:** Association between depressive symptoms at baseline and work nonparticipation at follow-up among females (n = 1245)

Outcome	Model 2^a^				
	Depressive symptoms				
	No (N = 690)	Mild (5–9) (n = 435)		Medium to severe (≥ 10) (n = 120)	
	OR	OR ^a^	95% CI	OR^a^	95% CI
TWNP	1	1.42	1.10–1.84	1.92	1.29–2.87
LTSA	1	1.56	1.18–2.07	2.57	1.69–3.91
UE	1	1.31	0.85–2.02	1.13	0.57–2.25
ER	1	1.13	0.68–1.89	1.05	0.46–2.40

TWNP: Total work nonparticipation; LTSA: Long-term sickness absence

UE: Unemployment; ER: Early Retirement

^a^ Adjusted for age, ISCO-skill level, paid employment at baseline

Among males, medium to severe depressive symptoms (OR = 3.22; 95% CI = 1.90–5.50) mild symptoms (OR = 1.59; 95% CI = 1.19–2.11) were significantly associated with TWNP. Both mild depressive symptoms (OR = 1.48; 95% CI 1.08–2.02) and medium to severe depressive symptoms (OR = 2.96; 95% CI = 1.76–4.97) were associated with LTSA. Medium to severe depressive symptoms were also associated with UE (OR = 5.17; 95% CI = 2.58–10.36). Mild depressive symptoms were significantly associated with ER (OR = 1.72; 95% CI = 1.01–2.92), while medium to severe symptoms were not.

Among females, medium to severe depressive symptoms were also associated with TWNP (OR = 1.92; 95% CI = 1.29–2.87) and with LTSA (OR = 2.57; 95% CI = 1.69–3.91). There was no significant association between depressive symptoms and both UE and ER.

### Depressive symptoms and duration of work nonparticipation

The second column in Tables 5 and 6 displays, for males and females respectively, the estimated number of months among those with no depressive symptoms (PHQ score 0–4) (Exp (β_0_)). Among males, there was a significant association between mild depressive symptoms and TWNP [Exp(β) = 1.4; 95% CI = 1.15–1.70]. The duration of TWNP was twice as long among participants presenting medium to severe depressive symptoms [Exp(β) = 2.06; 95% CI = 1.53–2.78]. The duration of LTSA was longer in both depressive symptoms groups [Mild: Exp(β) = 1.24; 95% CI = 1.02–1.51 – Medium to severe: Exp(β) = 1.78; 95% CI = 1.34–2.37]. The duration of ER was not significantly related to depressive symptoms. However, duration of UE was significantly associated with medium to severe depressive symptoms [Exp(β) = 1.94; 95% CI = 1.14–3.29].

**Table 5:** Baseline depressive symptoms and months of work nonparticipation during follow-up ^a^. N = 384 males

		Depressive symptoms				N
		Mild (5–9)		Medium to severe (≥ 10)		
Outcome	Exp (β_0_)^b^	Exp(β)^c^	95% CI	Exp(β)^c^	95% CI	
TWNP	5.39	1.40	1.15–1.70	2.06	1.53–2.78	384
LTSA	5.32	1.24	1.02–1.51	1.78	1.34–2.37	264
UE	6.27	1.51	0.98–2.34	1.94	1.14–3.29	88
ER	12.82	1.20	0.82–1.77	1.31	0.62–2.80	83

TWNP: Total work nonparticipation; LTSA: Long-term sickness absence; UE: Unemployment; ER: Early retirement

^a^ Among males experiencing work nonparticipation. The results of generalized linear models (GLMs)

^b^ Exp (β_0_) displays the estimated number of months among those with no depressive symptoms (PHQ score 0–4)

^c^ Adjusted for age, ISCO-skill level, paid employment at baseline

**Table 6:** Baseline depressive symptoms and months of work nonparticipation during follow-up ^a^. N = 458 females

		Depressive symptoms				N
		Mild (5–9)		Medium to severe (≥ 10)		
Outcome	Exp (β_0_)^b^	Exp(β)^c^	95% CI	Exp(β)^c^	95% CI	
TWNP	7.19	1.13	0.94–1.37	1.38	1.05–1.83	458
LTSA	5.85	1.30	1.07–1.57	1.49	1.15–1.94	313
UE	6.20	1.39	0.93–2.08	1.50	0.76–2.97	112
ER	45.18	0.76	0.50–1.16	1.47	0.72–3.02	85

TWNP: Total work nonparticipation; LTSA: Long-term sickness absence; UE: Unemployment; ER: Early retirement

^a^ Among females experiencing work nonparticipation. The results of generalized linear models (GLMs)

^b^ Exp (β_0_) displays the estimated number of months among those with no depressive symptoms (PHQ score 0–4)

^c^ Adjusted for age, ISCO-skill level, paid employment at baseline

Among females, the duration of TWNP was more than a third longer [Exp(β) = 1.38; 95% CI = 1.05–1.83] among participants presenting medium to severe depressive symptoms. The duration of LTSA was longer in both depressive symptoms groups [Mild: Exp(β) = 1.30; 95% CI = 1.07–1.57 – Medium to severe: Exp(β) = 1.49; 95% CI = 1.15–1.94]. Medium to severe depressive symptoms was not associated significantly with duration of ER and UE.

### Interactions by gender

Tests of interaction showed that, as a whole, the associations between depressive symptoms and events of work nonparticipation did not differ in the two genders. The only exception is the association between medium to severe depressive symptoms and UE, which was stronger among males than among females (p for interaction = 0.008; Males: OR = 5.17, 95% CI = 2.58–10.36; Females: OR = 1.13, 95% CI = 0.57–2.25).

Tests of interaction showed no gender differences in the associations between depressive symptoms and duration of work nonparticipation.

## Discussion

The present study suggests that medium to severe depressive symptoms are associated with subsequent TWNP, both in terms of event occurrence and duration. In addition, we found indications that also mild depressive symptoms are associated with subsequent TWNP, at least when it comes to the occurrence of events.

The present study suggests also significant associations between depressive symptoms and subsequent LTSA and UE. However, the associations with ER were not significant. With one exception only (i.e., events of UE, for which the associations were stronger among males), we did not find support for our expectation that the association of depressive symptoms with different forms of work nonparticipation would depend on gender.

### Comparison with other studies

To our knowledge, only a few previous longitudinal studies based on representative samples of employees have hitherto investigated associations between self-reported depressive symptoms and subsequent work nonparticipation [16–18, 20–22, 33]. Most of these studies were focused on sickness absence or LTSA [16–18, 21, 22]; two studies examined UE [20, 21], while another two focused on disability pension [20, 33]. Only one study used aggregated measures of work nonparticipation [21]. Most existing studies were conducted in Denmark, except for the SHARE study, which included a number of countries (mainly European) and was based on a sample of older employees [20], and two studies from Australia (HILDA) and the Netherlands (Maastricht cohort study) [16, 17].

Regarding TWNP, the aforementioned Danish study confirmed our finding that depressive symptoms were associated with a longer duration of TWNP, but only limited to LTSA and UE [21].

In relation to LTSA, our study confirmed previous findings from Australia [16], the Netherlands [17] and Denmark [18, 21, 22], all suggesting significant associations, similar in strength to the ones we observed, between depressive symptoms and LTSA [16–18, 21, 22]. Moreover, our findings are in line with two previous studies showing associations between mild depressive symptoms and LTSA [16, 18]. Two further studies confirmed the findings of the present study by suggesting a link between depressive symptoms and a longer duration of sickness absence [16, 21].

As for UE, our findings are in line with the aforementioned SHARE study and one of the mentioned studies from Denmark [20, 21].

We could not find any previous studies examining ER while combining disability pension and other types of ER as we did here. Two studies found associations with disability pension [20, 33], with hazard ratios indicating a risk that was two to three times higher among those with depressive symptoms.

As a whole, the limited available evidence about the possible associations between depressive symptoms and subsequent work nonparticipation makes it difficult to establish if the German situation deviates from that of other countries. In addition, measurements of depressive symptoms differ in the available studies. The instruments employed include the Major Depression Inventory (MDI) [18, 21], the Mental Health Inventory (MH-5) [16, 18, 22, 33], the EURO-D and the Hospital Anxiety and Depression scale HAD-D [17]. The question as to whether associations with work nonparticipation change as a function of how depression is measured is, therefore, still open.

### Strengths

One strength of the present study lies in the use of a longitudinal design, with depressive symptoms measured at baseline and employment biography followed up over the subsequent five years. The prevalence of depressive symptoms was captured in a representative sample of the German working population. The comparison of the sample characteristics with the referent population indicated no major selection bias at baseline. Additional strengths are the use of the PHQ-9 to measure depressive symptoms. This instrument is well established both in Germany and in international representative studies. With a cut-off value of > 9, it offers a case classification; in addition, the sum score provides a quantitative measure of depressive symptoms. From the perspective of prevention, it is important to recognize depressive symptoms before the full-blown disorder manifests. Supporting this, the present findings suggest that already mild depressive symptoms are associated with TWNP and LTSA.

A study comparing self-reports and register-based measures of LTSA, UE and ER over a longer time span suggests that self-reports are a valid tool to assess these phenomena [34]. Such data enable not only the recording of an event but also the quantification of its duration.

### Weaknesses

A first limitation is that no firm causal conclusions can be drawn based on the present findings. Features of our study design such as the measurement of the predictors prior to the measurement of the outcomes, as well as the inclusion of confounders through stratification and adjustment, are useful in supporting casual interpretations with regards to the examined associations [35]. Despite these features can reduce sources of bias, the present study remains an observational one, which limits conclusions about causality.

Second, participation in the cohort was low (18%). We did not observe differences in attrition due to gender, while differences due to age and skill level were moderate (see Method section). We believe that these differences in attrition did not introduce major biases in our risk estimates, considering that age and skill level were adjusted for in all analyses.

A third weakness might be that the study addresses only longer work nonparticipation events, as only events lasting months, instead of weeks or days, were considered. Additionally, only sickness absence episodes of at least 6 weeks were investigated. According to reports by the German Federal Ministry of Health, more than half of all recorded sick days relate to events with a duration below 42 days [3]. This could have led to a substantial under-recording of the total amount of sickness absence in our study. In the German context, the use of short-term sickness absence is limited as the German insurance system comprises a large number of different health insurance companies that use non comparable registration systems. As a result, we were unable to examine if depressive symptoms presented the same associations with subsequent short-term absence as with LTSA.

Fourth, the present study only considered the pathway from depressive symptoms to work nonparticipation, while reverse causation was not considered. In some cases, events of work nonparticipation such as UE might deteriorate mental health [36]; in other cases, events such as ER might relate to improved health [37]. Two-wave study designs are not sufficient to shed light on the processes, which require multi-wave studies [38]. Currently, the third wave of the S-MGA study is underway, which might provide a better basis for examining reversed causation.

Fifth, we adjusted for skill level and not for education in the regression analyses (see the covariates subsection of the Method section). As skill level and education were strongly correlated, adjusting for both could have led to unstable regression coefficients in light of the limited sample size as well as the low prevalence of depressive symptoms (Tables 2 and 1).

## Conclusions

From a clinical point of view, our study suggests that medium to severe depressive symptoms can lead to increased work nonparticipation. In addition, even mild-level depression can be predictive of increased work nonparticipation. Therefore, the early detection of possible cases of depression might contribute to higher participation in the labour market.

In light of the scarce evidence available, the present study is one of the first to suggest that depressive symptoms are a significant predictor of total work nonparticipation among both males and females. With regard to LTSA, UE and ER, our findings support the results of the few previous studies. Although administrative data on the diagnoses behind cases of sickness absence and disability pension are largely available, it is not known to what extent these diagnoses reflect a role of depressive symptoms in relation to sickness absence and disability pension in working populations. In a similar vein, it is not known what role depressive symptoms play relative to other types of pensions and to UE. To understand the possible impact of depressive symptoms, approaches quantifying associations with subsequent work nonparticipation are needed that go beyond the calculation of rate ratios (or hazard ratios/odds ratios) for the simple occurrence of events. In the present study, the adoption of two-part models represents an advancement as it considers not only the incidence or prevalence of nonparticipation events but also their duration. This approach could be relevant for the assessment of the economic consequences of depressive symptoms on participation in working life. When examining such consequences, it is important to take all types of work nonparticipation into account to reduce effect underestimation.

## Data Availability

A scientific use file (SUF) containing both wave 1 and wave 2 of the cohort is available at the Research Data Centre of the Federal Institute of Occupational Safety and Health. Acknowledgements: The longitudinal Study on Mental Health at Work (S-MGA) was conducted in collaboration with the Institute for Employment Research (IAB). The S-MGA was based on samples from statistics of the Federal Employment Agency (BA), which have been merged with Integrated Employment Biographies by the IAB. A scientific use file of the first wave and second wave data is available at the BAuA (DOI: 10.48697/smga.w1w2.suf).

## References

[1] Lagerveld S, Bültmann U, Franche R, Van Dijk F, Vlasveld M, Van der Feltz-Cornelis C, Bruinvels D, Huijs J, Blonk R, Van Der Klink J (2010). Factors associated with work participation and work functioning in depressed workers: a systematic review. J Occup Rehabil.

[2] Knieps F, Pfaff H (2020). BKK Gesundheitsreport 2019.

[3] Bundesgesundheitsministerium. Arbeitsunfähigkeit: Fälle und Tage nach Falldauer 2019 - Ergebnisse der Krankheitsartenstatistik der gesetzlichen Krankenversicherung. In.: BMG; 2019.

[4] Deutsche Rentenversicherung Bund (2019). Rentenversicherung in Zeitreihen.

[5] Schofield DJ, Shrestha RN, Percival R, Passey ME, Callander EJ, Kelly SJ (2011). The personal and national costs of mental health conditions: impacts on income, taxes, government support payments due to lost labour force participation. BMC Psychiatry.

[6] Pedersen J, Solovieva S, Thorsen SV, Andersen MF, Bultmann U. Expected Labor Market Affiliation: a New Method Illustrated by estimating the impact of perceived stress on time in work, sickness absence and unemployment of 37,605 Danish employees. Int J Environ Res Public Health 2021, 18(9). 10.3390/ijerph18094980 PMC8124718 34067104

[7] Buchholz S, Rinklake A, Blossfeld H-P. Reversing early retirement in Germany a longitudinal analysis of the effects of recent pension reforms on the timing of the transition to retirement and on pension incomes. Comp Popul Stud 2013, 38(4).

[8] Johansen K, Andersen JS, Mikkelsen S, Lynge E (2011). Decision making and co-operation between stakeholders within the process of sick leave. A case study in a Danish municipality. J Interprof Care.

[9] Maeland S, Werner EL, Rosendal M, Jonsdottir IH, Magnussen LH, Lie SA, Ursin H, Eriksen HR (2013). Sick-leave decisions for patients with severe subjective health complaints presenting in primary care: a cross-sectional study in Norway, Sweden, and Denmark. Scand J Prim Health Care.

[10] Bretschneider J, Janitza S, Jacobi F, Thom J, Hapke U, Kurth T, Maske UE (2018). Time trends in depression prevalence and health-related correlates: results from population-based surveys in Germany 1997–1999 vs. 2009–2012. BMC Psychiatry.

[11] Wittchen HU, Jacobi F, Rehm J, Gustavsson A, Svensson M, Jönsson B, Olesen J, Allgulander C, Alonso J, Faravelli C (2011). The size and burden of mental disorders and other disorders of the brain in Europe 2010. Eur Neuropsychopharmacology: J Eur Coll Neuropsychopharmacol.

[12] Wittchen HU (1994). Reliability and validity studies of the WHO–Composite International Diagnostic interview (CIDI): a critical review. J Psychiatr Res.

[13] Wittchen H-U, Pfister H. DIA-X-interviews: manual für screening-Verfahren und interview; Interviewheft. 1997.

[14] Beck AT, Ward CH, Mendelson M, Mock J, Erbaugh J (1961). An inventory for measuring depression. Arch Gen Psychiatry.

[15] Hautzinger M, Keller F, Kühner C. Beck depressions-inventar (BDI-II). Harcourt Test Services; 2006.

[16] Wooden M, Bubonya M. Cobb-Clark DJSJoW, Environment, Health: sickness absence and mental health: evidence from a nationally representative longitudinal survey. 2016(3):201–8. 10.5271/sjweh.3553 26881765

[17] Lexis MAS, Jansen NWH, van Amelsvoort LGPM, van den Brandt PA, Kant I. Depressive complaints as a predictor of sickness absence among the Working Population. 2009, 51(8):887–95. 10.1097/JOM.0b013e3181aa012a 19625974

[18] Thorsen SV, Rugulies R, Hjarsbech PU, Bjorner JB (2013). The predictive value of mental health for long-term sickness absence: the Major Depression Inventory (MDI) and the Mental Health Inventory (MHI-5) compared. BMC Med Res Methodol.

[19] van Rijn RM, Robroek SJ, Brouwer S, Burdorf A (2014). Influence of poor health on exit from paid employment: a systematic review. Occup Environ Med.

[20] Porru F, Burdorf A, Robroek SJW (2019). The impact of depressive symptoms on exit from paid employment in Europe: a longitudinal study with 4 years follow-up. Eur J Public Health.

[21] Pedersen J, Thorsen SV, Andersen MF, Hanvold TN, Schlünssen V, Bültmann U (2019). Impact of depressive symptoms on worklife expectancy: a longitudinal study on Danish employees. Occup Environ Med.

[22] Bültmann U, Rugulies R, Lund T, Christensen KB, Labriola M, Burr H (2006). Depressive symptoms and the risk of long-term sickness absence. Soc Psychiatry Psychiatr Epidemiol.

[23] Bultmann U, Christensen KB, Burr H, Lund T, Rugulies R (2008). Severe depressive symptoms as predictor of disability pension: a 10-year follow-up study in Denmark. Eur J Pub Health.

[24] Kausto J, Pentti J, Oksanen T, Virta LJ, Virtanen M, Kivimäki M. Vahtera JJSJoW, Environment, Health: length of sickness absence and sustained return-to-work in mental disorders and Musculoskeletal Diseases: a cohort study of public sector employees. 2017(4):358–66. 10.5271/sjweh.3643 28463382

[25] Nieuwenhuijsen K, Verbeek JHAM, de Boer AGEM, Blonk RWB, van Dijk FJH (2006). Predicting the duration of sickness absence for patients with common mental disorders in occupational health care. Scand J Work Environ Health.

[26] Thorsen SV, Pedersen J, Flyvholm M-A, Kristiansen J, Rugulies R, Bültmann U (2019). Perceived stress and sickness absence: a prospective study of 17,795 employees in Denmark. Int Arch Occup Environ Health.

[27] Rose U, Schiel S, Schröder H, Kleudgen M, Tophoven S, Rauch A, Freude G, Müller G (2017). The study on mental health at work: design and sampling. Scand J Public Healt.

[28] d’Errico A, Burr H, Pattloch D, Kersten N, Rose U (2021). Working conditions as risk factors for early exit from work—in a cohort of 2351 employees in Germany. Int Arch Occup Environ Health.

[29] Löwe B, Spitzer RL, Zipfel S, Herzog W (2002). PHQ-D. Gesundheitsfragebogen für Patienten.

[30] Manea L, Gilbody S, McMillan D (2015). A diagnostic meta-analysis of the Patient Health Questionnaire-9 (PHQ-9) algorithm scoring method as a screen for depression. Gen Hosp Psychiatry.

[31] Min Y, Agresti A (2002). Modeling nonnegative data with clumping at zero: a survey. J Iran Stat Soc.

[32] Smyth GK. Regression analysis of quantity data with exact zeros. In: *Proceedings of the second Australia–Japan workshop on stochastic models in engineering, technology and management: 1996*: Citeseer; 1996: 572–580.

[33] Bültmann U, Christensen KB, Burr H, Lund T, Rugulies R (2008). Severe depressive symptoms as predictor of disability pension: a 10-year follow-up study in Denmark. Eur J Public Health.

[34] Wahrendorf M, Marr A, Antoni M, Pesch B, Jöckel K-H, Lunau T, Moebus S, Arendt M, Brüning T, Behrens T (2018). Agreement of self-reported and Administrative Data on Employment histories in a German cohort study: a sequence analysis. Eur J Popul.

[35] Little RJ, Rubin DB (2000). Causal effects in clinical and epidemiological studies via potential outcomes: concepts and analytical approaches. Annu Rev Public Health.

[36] Demiral Y, Ihle T, Rose U, Conway PM, Burr H (2022). Precarious work as risk factor for 5-year increase in depressive symptoms. Int J Environ Res Public Health.

[37] Westerlund H, Kivimäki M, Singh-Manoux A, Melchior M, Ferrie JE, Pentti J, Jokela M, Leineweber C, Goldberg M, Zins M (2009). Self-rated health before and after retirement in France (GAZEL): a cohort study. Lancet.

[38] Taris TW, Kompier MA. Cause and effect: Optimizing the designs of longitudinal studies in occupational health psychology. In., vol. 28: Taylor & Francis; 2014: 1–8.

